# A ppm Ethanol Sensor Based on Fabry–Perot Interferometric Surface Stress Transducer at Room Temperature

**DOI:** 10.3390/s20236868

**Published:** 2020-11-30

**Authors:** Toshiaki Takahashi, Yong-Joon Choi, Kazuaki Sawada, Kazuhiro Takahashi

**Affiliations:** 1 Department of Electrical and Electronic Information Engineering, Toyohashi University of Technology, Toyohashi, Aichi 441-8580, Japan; takahashi.toshiaki.zl@tut.jp (T.T.); choi@ee.tut.ac.jp (Y.-J.C.); kazuaki.sawada@tut.jp (K.S.); 2 Japan Society for the Promotion of Science, Tokyo 102-0083, Japan; 3 Electronics Inspired-Interdisciplinary Research Institute (EIIRIS), Toyohashi University of Technology, Toyohashi, Aichi 441-8580, Japan

**Keywords:** fabry–perot interference, microelectromechanical systems, surface stress sensor, optical interferometry, film transfer technique, volatile organic compounds, ethanol, chemical sensing

## Abstract

Disease screening by exhaled breath diagnosis is less burdensome for patients, and various devices have been developed as promising diagnostic methods. We developed a microelectromechanical system (MEMS) optical interferometric surface stress sensor to detect volatile ethanol gas at room temperature (26~27 °C) with high sensitivity. A sub-micron air gap in the optical interferometric sensor reduces interference orders, leading to increased spectral response associated with nanomechanical deflection caused by ethanol adsorption. The sub-micron cavity was embedded in a substrate using a transfer technique of parylene-C nanosheet. The sensor with a 0.4 µm gap shows a linear stable reaction, with small standard deviations, even at low ethanol gas concentrations of 5–110 ppm and a reversible reaction to the gas concentration change. Furthermore, the possibility of detecting sub-ppm ethanol concentration by optimizing the diameter and thickness of the deformable membrane is suggested. Compared with conventional MEMS surface stress gas sensors, the proposed optical interferometric sensor demonstrated high-sensitivity gas detection with exceeding the detection limit by two orders of magnitude while reducing the sensing area.

## 1. Introduction

The biomarkers in the breath and blood are an index for identifying the existence and degree of progression of various diseases [1,2]. If these biomarkers, including gas molecules and biomacromolecules, can be detected from minimal traces of specimens without labeling, it will become possible to diagnose diseases easily, rapidly, and inexpensively in areas with few medical staff and small clinics. The need for such point-of-care testing (POCT) devices is rising every year, and since the global market for POCT devices is expected to grow to 46.7 billion dollars by 2024 [3], such device development has been attracting increased attention.

Cancer-derived protein markers such as prostate specific antigen (PSA) and carcinoembryonic antigen (CEA) are used in practice as a marker screening technique. As a less invasive screening technique, volatile organic compounds (VOCs) in exhaled breath act as biomarkers for diagnosing diabetes and renal failure, lung cancer, and have attracted attention in recent years as a promising diagnostic method for diseases with low patient burden [4,5,6,7]. While the concentration of volatile ethanol gas in the exhaled breath is reported at 27–216.1 ppb in healthy people, it increases to 64−2160 ppb in patients of lung cancer and the average value of them is 0.6 ppm [8]. Therefore, it can be used for patient screening by achieving an operating range of ppm to sub-ppm. In recent years, various sensors have been developed to detect ethanol (EtOH), a kind of VOC. Y. Zhang et al. have succeeded in detecting a wide dynamic range of 0.001–1000 ppm with high sensitivity in a gas sensor based on SnO_2_ films, as a method to read out the resistance change caused by target molecules adsorbed on the gas-reactive film [9]. Because the oxide semiconductor-based gas-reactive film must be heated up to several hundred degree celsius for the sensing operation, the heater must be integrated around the sensing part, leading to increased power consumption. Assuming that it will be used as Internet of Things (IoT) gas sensors, it will be a big issue. For reducing the heater power consumption, the sensing area is released from the substrate through a hole, decreasing the heating capacity of the sensing area and preventing temperature rise in peripheral circuits [10,11]. However, issues such as the complexity of the fabrication process from integrating heaters into a sensor and an increase in footprint per unit element due to isolated heating parts from the peripheral circuits persists. It is considered that discrimination between gas species without high selectivity of gas-reactive film is possible. Therefore, in recent years, gas sensors operated at room temperature without heaters have attracted much attention.

Semiconductor-based gas sensors have been reported to operate even at room temperature, but the limit of detection (LOD) decreases to about several ppm [12,13]. As an another detection method which can operate at room temperature, in the sensor using ZnO_2_ nanohybrid thin film as a gas-reactive film, they have succeeded in detecting ethanol gas at a concentration range of 10–100 ppm by readouting the amount of change in effective refractive index as a change in peak shift of reflection spectrum when white light is irradiated [14]. Alternatively, a microelectromechanical system (MEMS) surface stress sensor, a cantilever-based nanomechanical biosensor [15,16,17], has been reported as a gas sensor for room temperature operation. The MEMS-based gas sensor readouts the nanomechanical deflection associated with the adsorption of a target molecule as a piezoresistive change [18,19,20]. An epoxy acrylate film with a gas-reactive film was used to successfully detect EtOH in a wide dynamic range of 200–16,000 ppm [18]. Despite the challenges of detection sensitivity, surface stress sensors have been widely studied as bio and gas sensors because they can be used to detect various target molecules by selecting the appropriate reactive membrane. In general, although gas-reactive films have an issue of the selectivity of gases, gas species can be identified by acquiring the response patterns of several types of gas-reactive films and implementing machine learning [19]. Therefore, sensor arrays and the coating of several reactive films are practical solutions.

Considering that the above-mentioned sensors are used for both bio and chemical sensing applications, surface stress sensors are the most versatile because they can be used in room temperature environments and can respond to bio and gas molecules by changing the adsorption layer (adsorbed receptor molecules). To improve the sensitivity of surface stress sensors, we develop a novel MEMS surface stress sensor that combines a Fabry–Perot interferometer and photodiode to increase the signal conversion efficiency of the membrane deflection to an electrical signal using optical interferometry. The fabricated MEMS optical interferometric sensor demonstrates high sensitivity to detecting target molecules in liquids using antigen–antibody reaction by improving the wavelength selectivity of the interferometer and surface stress sensitivity of the deformable membrane using soft materials [21,22]. We also develop an optical interferometric sensor integrated with a source follower circuit to obtain the sensor response as a voltage signal [23]. By depositing a film that reacts to the hydroxy groups contained in the alcohol, the sensor can detect volatile EtOH and serve as biosensors.

In this paper, we report the design, fabrication, and characterization of the optical interferometric surface stress gas sensor that detects volatile EtOH at room temperature (26~27 °C). By optimizing the optical design of the interferometer, the response characteristics were amplified, resulting in the ppm level detection of VOCs. In addition, the optical design of the interferometer, which changes only vertical parameters, can reduce the sensing area of conventional surface stress gas sensors by more than two orders of magnitude. To facilitate the deposition of gas-reactive membranes and molecular receptors toward a multi-detection platform, we developed a cavity-embedded structure in the substrate using the transfer process of parylene-C nanosheet.

## 2. Design and Simulation

### 2.1. Detection Principle

**Figure 1:**
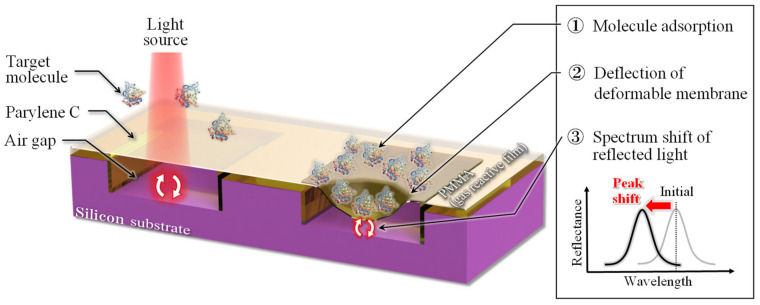
Schematic diagram and detection principle of microelectromechanical system (MEMS) optical interferometric surface-stress sensor.

Figure 1 shows the schematic diagram of the cross-sectional structure and detection principle of an optical interferometric surface stress sensor. The proposed sensor is composed of a parylene-C deformable membrane and gas-reactive polymethyl methacrylate (PMMA) layer on a cavity formed in a Si substrate; hence, the Fabry–Perot interferometer is comprised of the deformable PMMA/parylene-C membrane, air gap, and Si substrate. When a target gas molecule is absorbed in the gas-reactive layer, the deformable membrane is subjected to compressive or tensile stress as the gas-reactive layer expands or contracts, respectively. The deformable membrane, including the gas-sensitive layer, is deformed upward in the former and downward in the latter. This mechanical deflection is observed as a peak shift in the reflection spectrum, which enables us to detect the small deflection of the membrane caused by the adsorption of target molecules. Because the amount of deflection of the surface stress sensor is inversely proportional to Young’s modulus of the material used for the moving part and thickness of the film [24], improvements in detection sensitivity are expected when using a soft material with low Young’s modulus and thin film thickness. Moreover, sensors based on optical interferometry can increase the shift in the interference spectrum at the time of membrane deflection by narrowing the air gap of the interferometer. As the detection performance can be improved without expanding the area of the device by controlling the above parameters, highly sensitive chemical sensing at room temperature can be achieved while compensating for the drawbacks of the piezoresistive sensor.

### 2.2. Analysis of the Spectral Shift Associated with Deflection of Deformable Membrane

**Figure 2:**
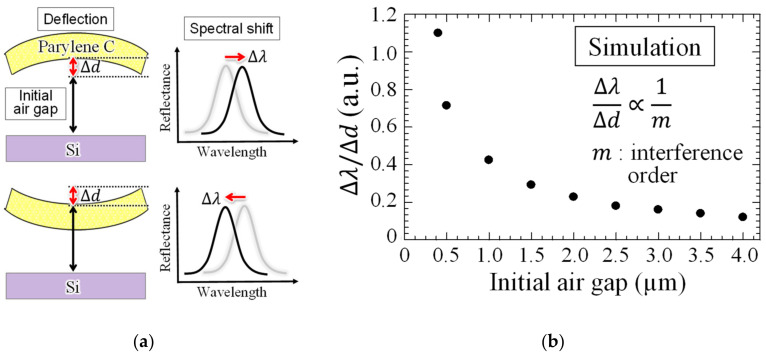
(**a**) Optical analysis model of the interferometer and (**b**) relationship between air-gap length of the interferometer and ratio of spectral shift to membrane deflection.

Figure 2 shows the optical analysis results of the ratio of the interference spectral shift associated with 30 nm deformation of the deformable membrane using optical analysis software (RSOFT DiffractMOD, Version 8.1.0.0.7, Synopsys Inc, USA). When the air-gap length of the interferometer is formed with the sub-micron scale, the peak shift involved with the membrane deflection increases. This means that the peak shift increases even when the deflection remains the same. Because the spectral shift ∆λ is inversely proportional to the interference order, the response is expected to be improved by narrowing the air gap by reducing the interference order to the visible region. In contrast, if the air gap is narrowed, the problem of stiction of the deformable membrane to the Si substrate occurs easily. Therefore, for preventing the stiction, we fabricate an interferometer with an air gap of 0.4 µm, which is sufficient for deflection during gas exposure. For validating the improvements, interferometers with air-gap lengths of 0.8 μm and 2.6 μm are also fabricated.

### 2.3. Analysis of Surface Stress Sensitivity of Deformable Membrane

**Figure 3:**
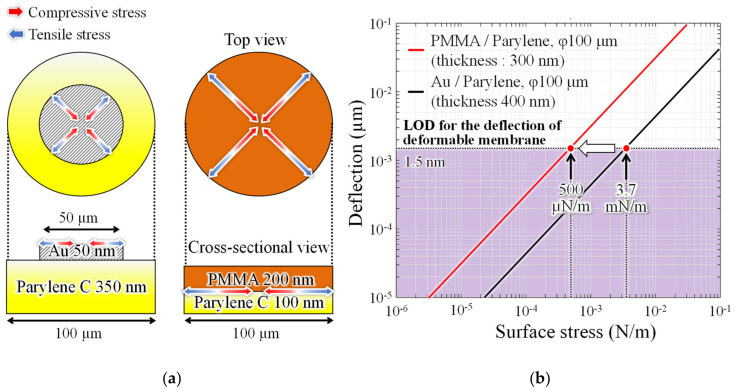
(**a**) Mechanical analysis model using the finite element method and (**b**) comparison of surface stress sensitivity between the conventional and proposed interferometer.

Figure 3 shows the analysis model, and membrane deflection results with surface stress applied to the deformable membrane using the finite element method. When PMMA expands due to gas adsorption, compressive stress is applied from the edge to the center of parylene-C. During contraction, tensile stress is applied in the opposite direction. The former is indicated as a red arrow and the latter as a blue arrow in Figure 3a. The analysis results in Figure 3b shows that the sensitivity of the deformable membrane to surface stress is larger when the deflection is smaller. The PMMA employed as a gas-reactive film has the following advantages: ease to form by spin coating and low Young’s modulus, which is one order magnitude lower than that of metallic films such as Au, commonly used as a molecular adsorption layer in biosensing [25,26]. In this design, the total film thickness, including PMMA and parylene-C layers, is set to 300 nm, which is reduced by 100 nm compared to the conventional structure [21]. In addition, we changed the coverage ratio of the molecular adsorption layer on the top surface of the deformable membrane, extending the area to apply surface stress fourfold. Here, the wavelength resolution of the spectrometer (USB4000, Ocean optics, Dunedin, FL, USA) that evaluates spectral shift is 0.3 nm, and the interference spectra are subjected to a 5-point moving average process after the acquisition of the spectra. Therefore, the lower limit of the measurable spectral shift is 1.5 nm. Defining LOD as the value of the surface stress causing 1.5 nm deformation, the LOD of the proposed structure is expected to be 7.4 times higher than that of the conventional structure.

## 3. Fabrication

**Figure 4:**
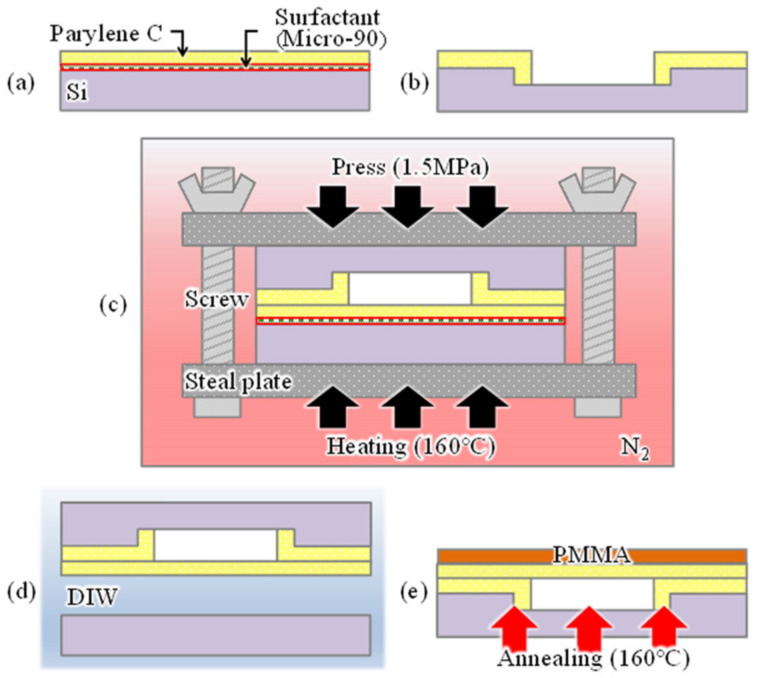
Fabrication process of the cavity-sealed interferometric transducer. (**a**) Deposition of parylene-C with surfactant, (**b**) patterning cavity and deposition of parylene-C, (**c**) bonding of two parts by thermal bonding, (**d**) releasing from bottom wafer by immersing in DIW, (**e**) annealing for improvement of adhesion between parylene layers, and deposition of PMMA.

Figure 4 shows the fabrication procedure of a cavity-sealed interferometric transducer. A strong bonding force is applied between the parylenes by simultaneously applying heat and pressure for a certain period with parylene-C layers in contact with each other to seal the cavity of the interferometer [27]. A parylene-C thin film deposited on a Si wafer coated with a surfactant (Micro-90, International Products Corp., Burlington, NJ, USA) (Figure 4a), and another parylene-C layer deposited on another Si wafer with a pre-formed cavity by reactive ion etching (Figure 4b) were prepared. Because the cavity depth formed in this process depends on the interferometer’s air-gap length, interferometers with different gap lengths of 0.4, 0.8, and 2.6 µm were formed to compare differences in the response. The diameters of their sensing areas were determined to be 100 µm. The pressure is applied in the two wafers by sandwiching them between steel plates, and applying torque with four screws. The plate area and screw specifications were selected so that the applying pressure becomes 1.5 MPa, similar to that reported in [27]. In addition, heat and pressure were simultaneously applied by heating at 160 °C for 10 min in an N_2_ gas atmosphere to bond the wafers (Figure 4c). The bonded wafer was immersed in deionized water, which reacts with the surfactant to peel off the Si substrate, leaving the parylene-C sheet on the Si wafer with cavities (Figure 4d). Annealing was then performed at 160 °C for 1 h to improve the adhesion between the transferred parylene-C sheet and parylene-C layer on the Si substrate (Figure 4e). The film thicknesses measured using the spectroscopic film thickness measurement system of the transferred and adhesive parylene-C were 98 nm and 180 nm, respectively.

**Figure 5:**
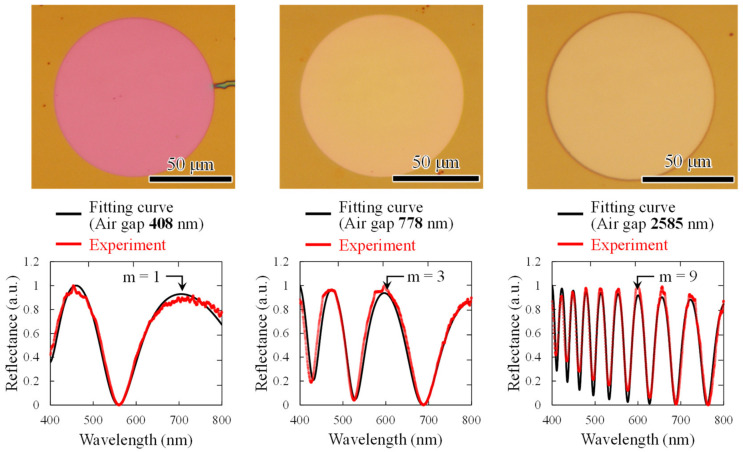
Optical microscope images (**top**) and reflection spectra (**bottom**) of developed Fabry–Perot interferometers. Fitting curves show good agreement with experimental values. The air gap is (**left**) 408 nm, (**center**), and (**right**) 2585 nm

Figure 5 shows optical microscope images of interferometers with different air gap lengths and results of spectroscopic measurements when white light is irradiated onto the interferometer. The interference order m was obtained as follows:

$$m=\frac{2}{\lambda }\left(n_{A}d_{A}+n_{P}d_{P}\right)$$

where *λ* is the wavelength of interference peak, *n_A_* and *n_P_* are the refractive indexes of air and parylene-C, respectively, *d_A_* is the air gap length, and *d_P_* is the thickness of parylene-C. A narrow air gap is found to reduce the interference order. The obtained spectra show good agreement with the analytical waveforms with the air-gap lengths of 408, 778, and 2585 nm, respectively, thereby successfully fabricating an interferometer close to the design values in sub-micro to micro scales.

## 4. Results and Discussion

**Figure 6:**
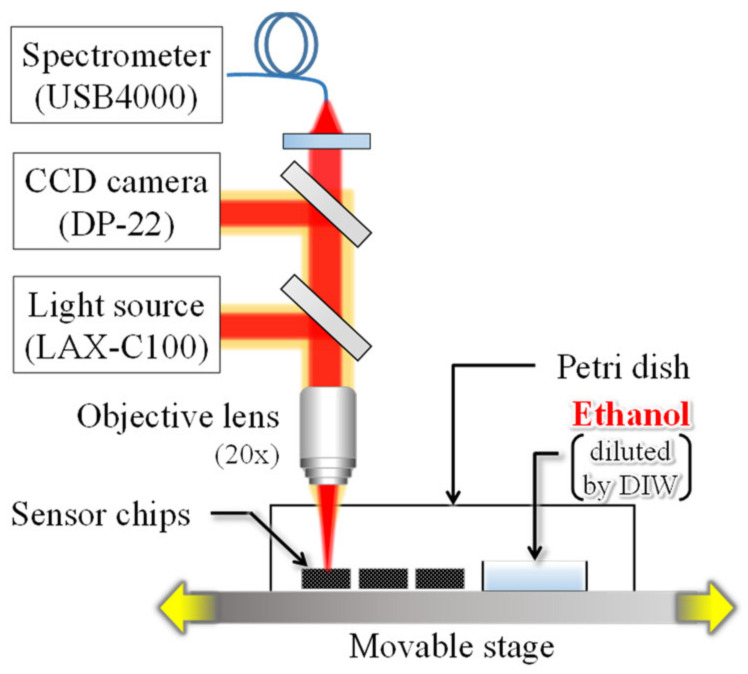
Schematic diagram of the experimental setup.

**Figure 7:**
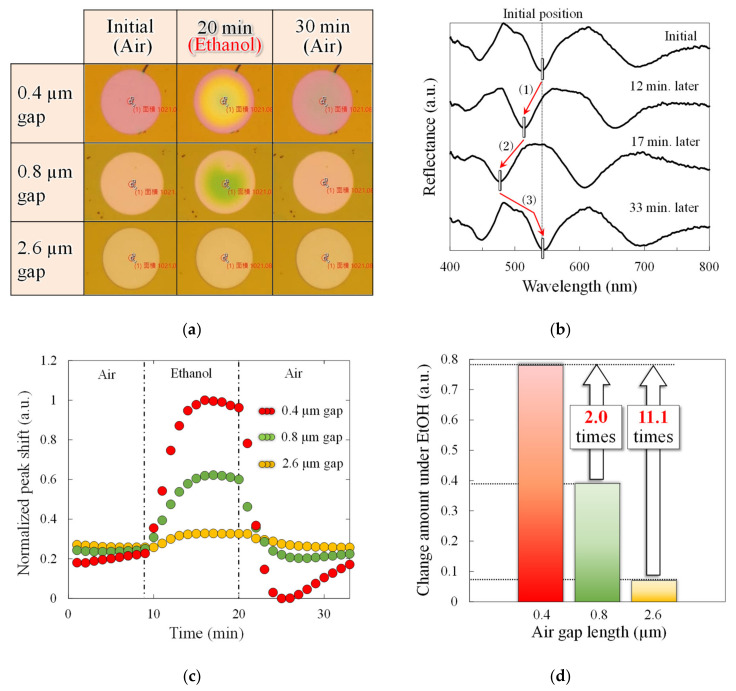
Comparison of interferometers with different air gaps under EtOH exposure. (**a**) Optical microscope images with color change associated with membrane deflection, (**b**) typical spectra shift in the interferometer of 0.8 µm gap, (**c**) change in peak shift to time, and (**d**) comparison of change amount in peak shift in the EtOH exposure.

To evaluate the performance improvements resulting from narrowing the gap length, PMMA was spin-coated onto interferometers with gap lengths of 0.4, 0.8, and 2.6 µm. The deflection of the deformable membrane upon exposure to EtOH gas was measured as a shift in the reflection spectrum. Figure 6 shows the detection system used in the experiment. Three interferometer chips with different gap lengths are arranged on a movable stage. The reflection spectrum, at 10 µm from the center of the deformable membrane, was acquired at 20-second intervals with white light irradiation. In addition, a small petri dish with 0.4 mL of EtOH solution (diluted with 50% DIW) was placed near the chips and sealed with a large petri dish to prevent leakage of the volatilized EtOH gas into the surroundings. The EtOH solution was placed in the vicinity of the chips for 9 to 20 min from the start of measurement and then removed. Figure 7 shows the response of the volatile EtOH gas to the sensor. As shown in Figure 7a, in the sub-micro scale narrow-gap interferometer, the membrane deflection is obtained as an interferometric color change. Note that the PMMA layer fixed to the substrate without the cavity does not change the interference color, which suggests that the change in refractive index due to EtOH adsorption in the PMMA layer is almost negligible. Figure 7b shows the time course of the reflection spectra in the interferometer with an air gap of 0.8 µm. After exposure to EtOH gas, the reflection spectrum blue shifts from (1) to (2). In other words, shortening the optical path length causes downward membrane deformation, suggesting that the absorption of EtOH causes PMMA film to shrink. Later, in the absence of exposure to the EtOH gas, the interference waveform red shifts to (3), resulting in overlaps with the interference waveform before the gas exposure. The results indicated that gas molecules reversibly adsorbed into the PMMA layer. In addition, the deformation direction suggests that the adsorbed molecules have an attractive force in the PMMA layer. Figure 7c shows the time course of the peak shifts associated with gas exposure in all interferometers. When the exposure to EtOH gas is stopped, the peak shift returns to the initial state, which means a reversible response due to the change in gas concentration is obtained in all interferometers. Figure 7d shows the maximum peak shift during gas exposure in all the interferometers. The change in the peak shift of the 0.4 µm gap interferometer with reduced interference order is 2.0 and 11.1 times higher than that of the 0.8 and 2.6 µm gap interferometers, respectively. The value of the 0.8 µm interferometer is approximately close to the optical analysis value of 2.1, while the value of the 2.6 µm interferometer is 1.8 times higher than the analysis value. This means that the 0.4 and 0.8 µm sensors have the same deflection amount during the gas response, while the 2.6 µm gap sensors have a reduced deflection. This may be due to the thickness of the PMMA film used as the gas-reactive film, which is thicker than that of the 0.4 µm gap interferometer. Because the surface stress sensitivity is inversely proportional to the square of the film thickness, the surface stress sensitivity is decreased by 1.8 times with a 35% thicker PMMA film in an interferometer with an air gap length of 2.6 µm. The PMMA layer deposited by spin coating may deform the parylene-C membrane downward due to the immediate pressure from dropping the liquid. Because the PMMA on the deformable membrane with a relatively deep cavity is locally formed thicker than other areas, it is assumed to have decreased the surface stress sensitivity of the 2.6 µm interferometer, thereby decreasing the membrane deflection. The narrow-gap interferometer demonstrated gas detection by the change in interference color with membrane deflection and improvements in spectral response.

**Figure 8:**
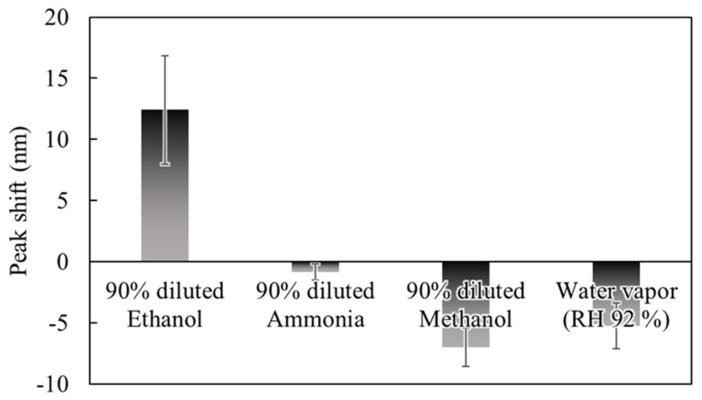
Comparison of gases response in interferometers with a 0.4 µm air gap and a 100 µm diameter. (Error bar means standard deviation of 3 interferometers on the same chip.)

To evaluate characteristics of the sensor response depending on gas species, we measured reflection spectra during exposure to 90% dilution of EtOH, ammonia, methanol (MeOH), and water vapor (92% relative humidity). Figure 8 shows the results of acquiring the peak shifts in the reflection spectrum of the sensor after exposure to 90% dilution of EtOH, ammonia, MeOH, and water vapor. The amount of peak shift on the vertical axis is positive for the direction in which the spectrum shifts to the shorter wavelength side. The largest peak shift was obtained for EtOH among the exposed gases, while exposure to gases other than EtOH resulted in a negative peak shift. In other words, since the deflection of the membrane occurs in the direction of cavity expansion, the PMMA layer of the gas-reactive membrane may absorb the gas and expand, resulting in compressive stress to the deformable membrane. Thus, the expansion rate of PMMA differs depending on the gas species, it is considered that there was a difference in the amount of peak shift. The result indicated that it is difficult to discriminate between gas species with a single device, but it is possible to discriminate between gas species by machine learning the differences in the response patterns of multiple gas-sensitive membranes [19]. Note that it was confirmed that the PMMA layer contracted and expanded in the case of absorption of EtOH and MeOH, respectively, which is helpful for gas species discrimination using machine learning.

**Figure 9:**
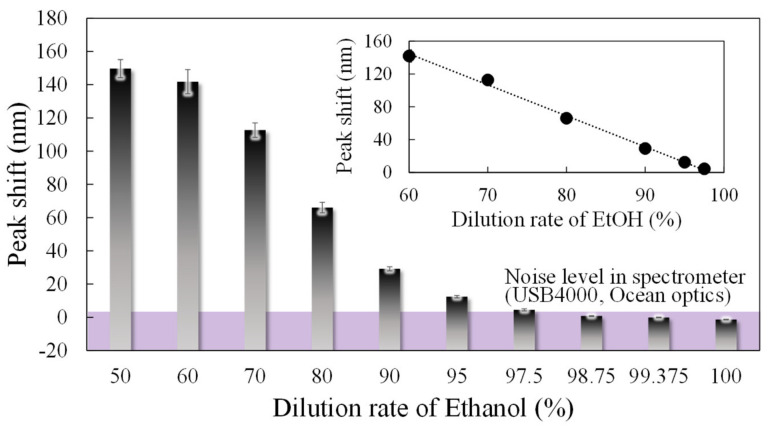
Concentration dependence under EtOH exposure in interferometers with a 0.4 µm air gap and a 100 µm diameter. (Error bar means standard deviation of 9 interferometers).

**Figure 10:**
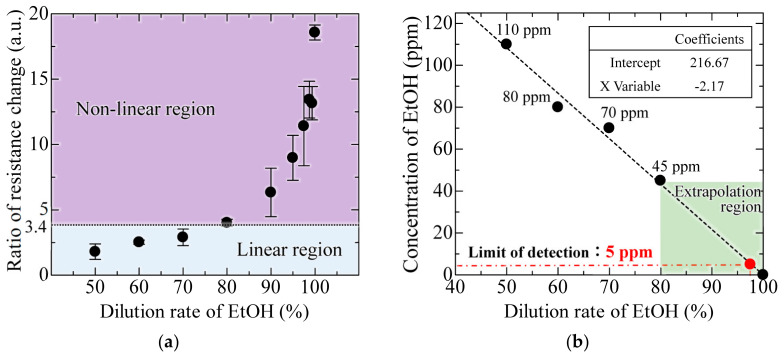
Estimation of the limit of detection (LOD) of EtOH in MEMS Interferometer using semiconductor gas sensor. (**a**) Output response of semiconductor gas sensor and (**b**) correlation between dilution rate and concentration of EtOH. (Error bar means standard deviation of 3 semiconductor gas sensors.).

To evaluate the concentration dependence of the 0.4 µm interferometer, which showed the highest response to ethanol in the above-mentioned experiments, we performed experiments to obtain the peak shifts with changes in the dilution rate of ethanol from 60 to 100%, as shown in Figure 9. The spectral response to changes in the ethanol dilution rate, on three interferometers with a 0.4 µm gap, was measured 9 times and is expressed as the standard deviation by the error bars. At a noise level of 1.5 nm, determined by the spectrometer and moving average processing, the shift amount of 4.4 nm at a dilution rate of 97.5% is the minimum LOD. In addition, the MEMS interferometer shows an approximately linear response when the dilution rate is between 60% and 97.5%. To obtain the correlation between the ethanol concentration and sensitivity, the concentration was identified with a commercial semiconductor gas sensor (TGS2620, FIGARO, Osaka, Japan). In Figure 10a, the semiconductor gas sensor shows a linear response in the range below 80% dilution rate, which is the guaranteed operating range represented by the ratio of resistance change of 0.18–3.4 [28]. In contrast, the stability is extremely degraded in the low concentration range, where the dilution rate exceeds 80%, although the response changes depending on the concentration. Figure 10b shows the result of estimating the concentration of EtOH from the obtained resistivity for dilution rate below 80%, showing a linear response in the semiconductor sensor. Assuming the EtOH concentration of 0 ppm at 100% dilution, and extrapolating the linear response region of the semiconductor gas sensor, the concentration of EtOH at 97.5% dilution was obtained as 5 ppm, which is the lower LOD of the MEMS interferometer. Furthermore, in the low concentration range (dilution rate of 90–97.5%) outside the guaranteed operation range of the commercial gas sensor, a stable response can be obtained with a standard deviation of 0.62–1.24 compared to 1.72–3.03 of the semiconductor sensor.

**Figure 11:**
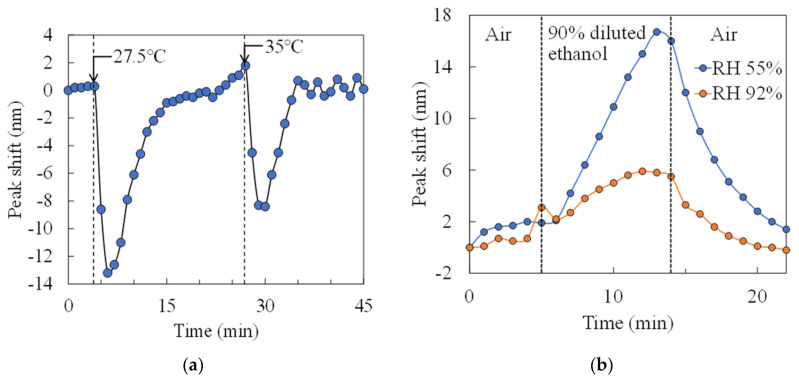
Impact of changes in temperature and humidity in interferometers with a 0.4 µm air gap and a 100 µm diameter. Time course of peak shift with (**a**) temperature change and (**b**) humidity change.

We measured the effects of temperature and humidity changes on the sensor. In the condition of no ethanol in petri dish, Figure 11a shows the time course of the peak shift when the temperature is changed by a hot plate. The sensor chip was heated at 20 °C for the first 5 minutes, 27.5 °C for 5 to 22 minutes, and 35 °C for 22 to 45 minutes in this experiment. Immediately after heating, the peak shift shows a negative value by the expansion of the air gap due to thermal expansion, while the peak position returned to its original position with time course. Therefore, the deflection of the membrane due to temperature changes can be solved by aging. Figure 11b shows the time course of the peak shift of 90% diluted ethanol exposure at a temperature of 20 °C and relative humidity of 55% and 92% (measured by a humidity sensor (HS1101LF, TE Connectivity)), respectively. The amount of change in the peak shift decreased under high humidity conditions while nanomechanical response was obtained even in the high humidity environment. According to this result, when the relative humidity changes from 55% to 92%, the peak shift amount decreases by about 63%. It is reported that the output response decreases by 50% or higher when the relative humidity changes from 50% to 90% in a semiconductor-based gas sensor using a heater [29]. Thus, the reduction in response in high humidity environments is same level to other gas sensors.

**Figure 12:**
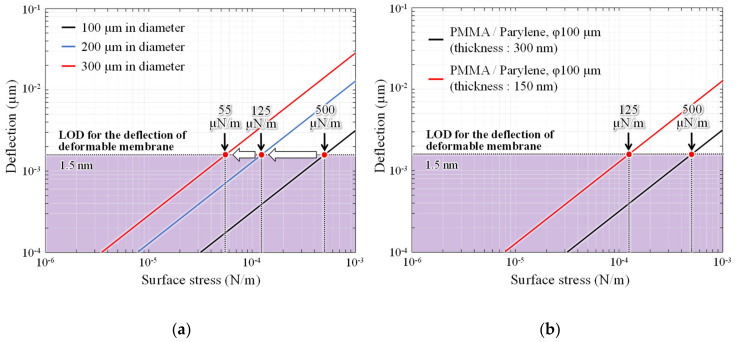
Improvement of detection limits by optimizing the surface stress sensitivity of interferometers. In the case of (**a**) expanding the diameter and (**b**) thinning of the deformable membrane.

For further improvements in minimum LOD, the sensitivity of the MEMS surface stress sensor was adjusted by changing the membrane diameter and thickness. Figure 12a shows changes in the surface stress sensitivity when the diameter of the deformable membrane is expanded from 100 µm to 200 and 300 µm. The surface stress sensitivity increases by ninefold when the diameter is extended by a factor of three.

The deformable membrane is further thinned without expanding the diameter. The surface stress sensitivity of the interferometers with 50-nm-thick parylene-C and 100-nm-thick PMMA, which are the minimum thicknesses formed by dry transfer and spin coating, respectively, is then measured. In this case, the surface stress sensitivity improves by approximately fourfold, which is equivalent to 125 µN/m the analysis value of sensitivity when the diameter is doubled. Therefore, the detection limit is expected to be improved by more than one order of magnitude, by increasing the area of the deformable membrane and thinning the thickness simultaneously. The sensing area size and LOD of the ethanol sensors operated under room temperature are summarized in Table 1. The fabricated interferometer area is more than two orders of magnitude smaller than that of the conventional piezoresistive surface stress sensor, and has superior detection limits. The sub-micron gap interferometric surface stress sensor presented here demonstrate ppm level gas detection, which is almost equivalent to the performance of the latest semiconductor-based gas sensor at room temperature. By optimizing the geometry parameters, a sensor capable of detecting sub-ppm ethanol concentrations can be developed, which exceeds the detection performance of conventional room-temperature gas sensors.

## 5. Conclusions

We developed an optical interferometry-based surface stress gas sensor to detect volatile EtOH gas at room temperature through nanomechanical deflections caused by gas adsorption in a deformable sub-micron-thick membrane. To improve the sensor response to the volatile EtOH gas, we fabricated a novel interferometer that can increase spectrum shift and stress sensitivity by narrowing the air gap and thinning the interferometer’s deformable membrane. Compared to a commercial semiconductor-based gas sensor, the fabricated interferometer, with a diameter of 100 µm and an air gap of 0.4 µm, demonstrated a linear response to EtOH concentrations of 5–110 ppm, and a stable readout signal with a small standard deviation in the low concentration ranges. Although the optical design has been optimized in this paper, the feasibility of a sub-ppm detection sensor was suggested by optimizing the mechanical parameters, such as the diameter and thickness of the deformable membrane. The lower LOD shown here achieved the best characteristics among unheated ethanol sensors. The proposed interferometer can serve as a highly sensitive multi-molecular gas- and bio-sensor by modifying the reactive layer and receptors on the deformable membrane.

## Figures and Tables

**Table 1:** Comparison of EtOH sensing reports at room temperature.

Device Type	Sensing Area (µm^2^)	LOD for EtOH (ppm)	LOD for Surface Stress (µN/m)	Ref.
SnO_2_–rGO hybrid film	N/A	1	N/A	[13]
Piezoresistive cantilever	1.2 × 10^6^ (Si bridge structure, 1.1 mm ×1.1 mm)	200	N/A	[18]
(a) This study (Experiment)	7.9 × 10^3^ 100 µm in diameter, 300 nm in thickness (150x smaller than the piezoresistive type)	5	500	N/A
(b) This study (Analysis)	2.4 × 10^4^ 300 µm in diameter, 300 nm in thickness (50x smaller than the piezoresistive type)	N/A	125	N/A
(c) This study (Analysis)	2.4 × 10^4^ 300 µm in diameter, 150 nm in thickness (50x smaller than the piezoresistive type)	N/A	55	N/A
